# A randomized controlled trial to assess the potential efficacy, feasibility and acceptability of an m-health intervention targeting parents of school aged children to improve the nutritional quality of foods packed in the lunchbox ‘SWAP IT’

**DOI:** 10.1186/s12966-019-0812-7

**Published:** 2019-07-02

**Authors:** Rachel Sutherland, Nicole Nathan, Alison Brown, Serene Yoong, Meghan Finch, Christophe Lecathelinais, Renee Reynolds, Alison Walton, Lisa Janssen, Clare Desmet, Karen Gillham, Vanessa Herrmann, Alix Hall, John Wiggers, Luke Wolfenden

**Affiliations:** 1 Hunter New England Population Health, Locked Bag 10, Wallsend, NSW 2287 Australia; 2 School of Medicine and Public Health, University of Newcastle, University Drive, Callaghan, NSW 2308 Australia; 3 Hunter Medical Research Institute, 1/Kookaburra Circuit, New Lambton Heights, NSW 2305 Australia; 4 Priority Research Centre for Health Behaviour, University of Newcastle, University Drive, Callaghan, NSW 2308 Australia

**Keywords:** Childhood obesity, Lunchboxes, Children, Child nutrition, M-health, Schools

## Abstract

**Background:**

Scalable interventions that improve the nutritional quality of foods in children’s lunchboxes have considerable potential to improve child public health nutrition. This study assessed the potential efficacy, feasibility and acceptability of an m-health intervention, ‘SWAP IT’, to improve the energy and nutritional quality of foods packed in children’s lunchboxes.

**Methods:**

The study employed a 2X2 factorial cluster randomized-controlled trial design**.** Twelve primary schools in New South Wales, Australia were randomly allocated to one of four groups: (i) no intervention;(ii) physical activity intervention only;(iii) lunchbox intervention only; or(iv) physical activity and lunchbox intervention combined. The two intervention strategies were evaluated separately. This paper focuses on the effects of the lunchbox intervention only. The lunchbox intervention comprised four strategies: 1) school nutrition guidelines; 2) lunchbox lessons; 3) information pushed to parents via a school-communication app and 4) parent resources addressing barriers to packing healthy lunchboxes. Outcome measures were taken at baseline and immediately post-intervention (10 weeks) and included measures of effectiveness (mean energy (kJ) packed in lunchboxes, total energy and percentage energy from recommended foods consistent with Australian Dietary Guidelines), feasibility (of delivering intervention to schools, parent app engagement and behaviour change) and acceptability to school staff and parents. Linear mixed models were used to assess intervention efficacy.

**Results:**

Of the 1915 lunchbox observations, at follow-up there was no significant differences between intervention and control group in mean energy of foods packed within lunchboxes (− 118.39 kJ, CI = -307.08, 70.30, *p* = 0.22). There was a significant increase favouring the intervention in the secondary outcome of mean lunchbox energy from recommended foods (79.21 kJ, CI = 1.99, 156.43, *p* = 0.04), and a non-significant increase in percentage of lunchbox energy from recommended foods in intervention schools (4.57%, CI = -0.52, 9.66, *p* = 0.08). The views of the messages pushed via the app ranged from 387 to 1550 views per week (mean views =1025 per week). A large proportion (71%) of parents reported awareness of the intervention, making healthier swaps in the lunchbox (55%), and pushed content was helpful (84%).

**Conclusion:**

The study is the first RCT to assess the potential of a multi-component m-health lunchbox intervention. The intervention was feasible, acceptable and potentially effective in improving the nutritional quality of foods packed within children’s lunchboxes.

**Trial registration:**

Australian Clinical Trials Registry ACTRN: ACTRN12616001228471.

## Background

Globally, almost 40% of adults and 25% of children are overweight or obese, resulting in 3.4 million deaths per year [1, 2] and an estimated global cost of $US2.0 trillion [3]. Poor dietary habits and low levels of physical activity are major contributors to the rising global burden of overweight and obesity [4]. Considering obesity in children and adolescents tracks into adulthood [5] obesity prevention in childhood is a global public health priority, requiring interventions across a number of settings, targeting individuals, organisations and community environments.

Consumption of foods high in energy but low in essential nutrients (‘discretionary’ foods), are a key driver of overweight and obesity as they provide excess energy and displace the intake of healthier foods [6, 7]. Schools are ideally positioned to deliver obesity prevention initiatives targeting children and families to reduce discretionary food intake given their access to large numbers of children and existing infrastructure to deliver interventions that influence children’s eating and dietary behaviours [8]. In countries such as Australia, New Zealand, the United Kingdom [9] and the United States [10] school lunchboxes, or sack lunches, are a significant source of food for children whilst at school, accounting, for up to a third of a child’s daily energy intake [11]. International research, however, suggests the provision of discretionary foods appear over represented in children’s lunchboxes [9, 11]. For example, studies with Australian primary school children reported that the average lunchbox contains over 3000 kilojoules (kJ) of foods and beverages, approximately 40% of an active primary school aged child’s total daily energy intake [12], and more than three serves of discretionary foods [13].

Few interventions have targeted the nutritional content of school lunchboxes. Intervention trials that have been undertaken have primarily focused on increasing the provision of water and/or nutrient rich foods such as fruit and vegetables [9, 14–16] without a corresponding focus on reducing discretionary foods. Similarly, previously trialled interventions have utilised strategies (e.g print materials) that have been ineffective in reaching or engaging large numbers of parents who are responsible for packing children’s lunches or have been delivered using modalities (face to face education) that are not amenable to delivery at scale [17, 18]. As such, a systematic review of lunchbox interventions have found that they have had limited impact in reducing the overall mean energy content of school lunchboxes [17].

Interventions utilising mobile applications (m-Health interventions) can be effective in improving health behaviours [19] and have the potential to reach large numbers of parents at low cost, representing a potentially scalable means of supporting the packing of healthy lunchboxes [19, 20]. School systems are increasingly using school communication apps to communicate directly with parents regarding school events, activities, policies, and student outcomes [21, 22]. Embedding interventions into these platforms may represent an effective way to improve the nutritional content of lunchboxes overcoming cited barriers faced by m-health interventions in securing sufficient reach and engagement by end-users. A recent cross sectional study investigating this proposition found that 80% of parents were interested in receiving support via an app to assist in packing healthy school lunchboxes [23]. In addition, a further study found 81% of principals believed it would be appropriate for lunchbox support to be delivered to parents via their school communication mobile app [22].

Despite this potential, utilisation of schools’ existing technological infrastructure has not yet been undertaken in previous trials of lunchbox interventions. As interventions targeting school lunchboxes delivered via this infrastructure have not previously been tested, formative pilot trials that can assess the feasibility and potential of such interventions are warranted. We sought to conduct such a trial to assess the potential efficacy, feasibility and acceptability of an m-health intervention to improve the energy and nutritional quality of foods packed in children’s lunchboxes. The potential impact on the mean total energy (kJ) from recommended foods (i.e. those foods that are low in saturated fat, added sugar and/or added salt, consistent with Guideline 2 and 3 of the Australian Dietary Guidelines [6]) and percentage energy from recommended foods packed in school children’s lunchboxes was assessed in addition to the intervention feasibility and acceptability from a school and parent perspective. The findings from the trial may provide evidence to support a larger randomized trial designed to assess longer-term effectiveness of such an intervention and impact on student dietary intake and weight status.

## Methods

### Ethics and registration

**Fig. 1:**
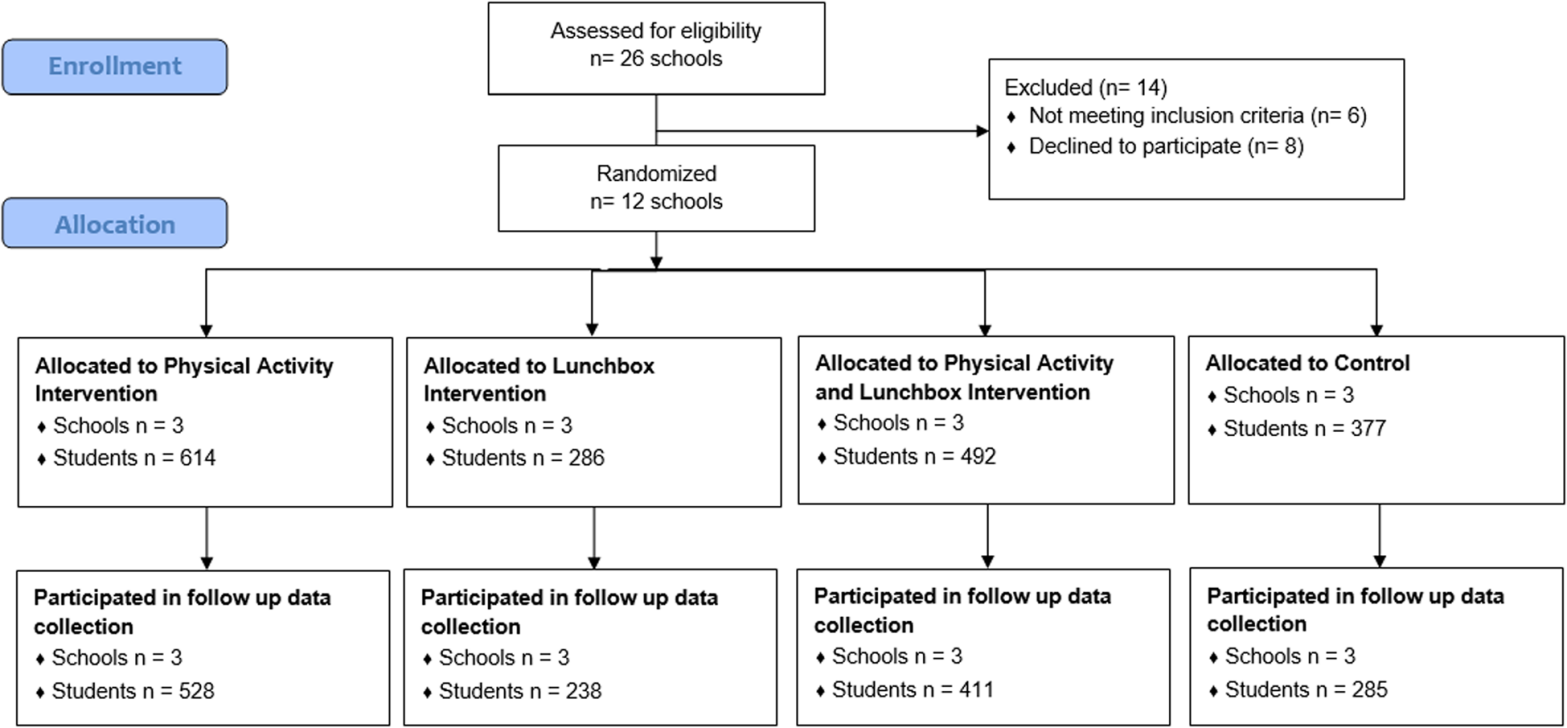
Consort flow diagram

Approval to conduct this study was obtained from Hunter New England Human Research Ethics Committee (Ref. No. 06/07/26/4.04), University of Newcastle (Ref. No. H-2008-0343), and the Maitland-Newcastle Catholic Schools Office and was prospectively registered with Australian New Zealand Clinical Trials Register ACTRN12616001228471 and follows the CONSORT reporting guidelines for pilot studies [24] (Fig. 1).

### Study design and setting

The study was conducted as a part of a broader 2X2 factorial cluster randomized controlled trial (RCT), which tested the impact of two initiatives including; (i) a physical activity intervention designed to support schools to increase the time scheduled for students to engage in moderate and some vigorous physical activity across the school week and; (ii) a lunchbox intervention designed to support parents to improve the nutritional quality of foods brought from home in lunchboxes for children to consume at school. The interventions were conducted as exploratory trials to determine their potential efficacy, feasibility and acceptability. Specifically, 12 Catholic primary schools (those catering for children aged 5–12 years) in the Hunter region of New South Wales (NSW), Australia were randomized to one of four treatment groups (see Fig. 1) i.e. (i) physical activity support only; (ii) lunchbox support only; (iii) combined physical activity and lunchbox support; or (iv) wait-list control. The factorial designed trial provided an efficient opportunity for the health service funder to test the impacts of the two interventions. Thus the factorial study design was used for efficiency, with an overall objective to assess separately the efficacy of the two interventions; it was not designed nor powered to evaluate an interaction effect between the two intervention strategies. While both physical activity and lunchbox measures were co-primary outcomes, they test independent interventions and were prospectively registered to be reported separately. Therefore, in this paper, we report the impact of the lunchbox intervention on nutrition (lunchbox outcomes) only, by comparing those who received the lunchbox intervention (i.e. those who received the lunchbox support only and those who received the combined physical activity and lunchbox support) to those who did not receive the lunchbox intervention (i.e. those who received the physical activity support only and those who received wait-list control).

Data were collected from consenting parents and students at baseline and immediately post intervention. The primary trial outcomes were the mean energy (kJ) content of foods packed in children’s lunchboxes, measured via observation and assessed using a validated school food checklist for assessment [25]. Secondary outcomes included the mean total energy (kJ) from recommended foods and percentage energy from recommended foods packed in school children’s lunchboxes, such as those encouraged within the Australian Dietary Guidelines. Intervention feasibility and acceptability of the research procedures and the intervention implementation were also assessed.

### Sample and participants

#### Schools

Primary schools from the study region were considered eligible for inclusion if they met the following criteria: Catholic school; had greater than 120 student enrolments; current user of the preferred school mobile communication app (required for the lunchbox treatment group); and were not participating in other nutrition or physical activity based research studies. Schools catering for students aged 13–18 years, schools catering for children with special needs (such as intellectual disabilities) and those participating in another physical activity intervention were excluded. School principals were provided with a study information package and asked to provide written informed consent. Recruitment continued until 12 schools consented to participate. Of the 26 schools randomly selected, 6 were ineligible as they did not have the school communication app or had participated in a previous pilot; 20 were approached and 12 agreed to participate. Across the participating schools, 3 schools were located in regions classified as regional or remote Australia and 8 were considered low SES. Three of the schools had greater than 10% of students that identified as being from Aboriginal or Torres Strait Islander background.

#### Students

All students in Kindergarten to grade 6 (ages 5 to 12 years) attending intervention schools were exposed to the intervention. Students from all schools were invited to take part in the data collection component of the trial. Students were invited to take part via an information package sent to their parents who were asked to provide active consent via a signed consent form. Parents were encouraged to discuss the study procedures with their child prior to consent. Two weeks following distribution of the information packages, parents who had not returned a consent form were telephoned by staff employed through the school and asked if their child could participate in study measurement. A replacement consent form was sent to parents providing verbal consent. Student assent was also required on the day of data collection. Data from students whose parents were not active users of the school app (identified as an app user on the child’s consent form) were excluded from analysis.

### Randomization and blinding

Following recruitment and baseline data collection, schools were randomly allocated in a 1:1:1:1 ratio to one of four groups (described above) by an independent investigator using a computerized random number function in Microsoft Excel. The parents of the primary schools randomly allocated to the lunchbox intervention arms received a multi-component 10 week lunchbox support intervention delivered primarily via an existing school mobile communication app or to a waitlist control. Data collector coordinators and data collectors were blinded to group allocation at baseline however at follow-up, the data collection coordinators were aware of group allocation. Due to the nature of the intervention, school staff were aware of their group allocation.

### Intervention development

#### Conceptual framework

The intervention, titled “SWAP IT”, was developed to be consistent with the World Health Organization’s (WHO) Health Promoting Schools (HPS) framework that recommends school based health promotion interventions include strategies that address the school curriculum, school environment and community [26–30]. The specific components and behaviour change strategies employed by the intervention were developed and selected using the Behaviour Change Wheel (BCW) [31], a comprehensive framework that draws on 19 different theories of behaviour change providing a comprehensive framework for intervention development [31]. Specifically, to identify the key modifiable determinants of packing a healthy lunchbox and to understand the context, the research team undertook reviews of the scientific literature, focus groups with parents; and telephone interviews with parents and school principals.

**Fig. 2:**
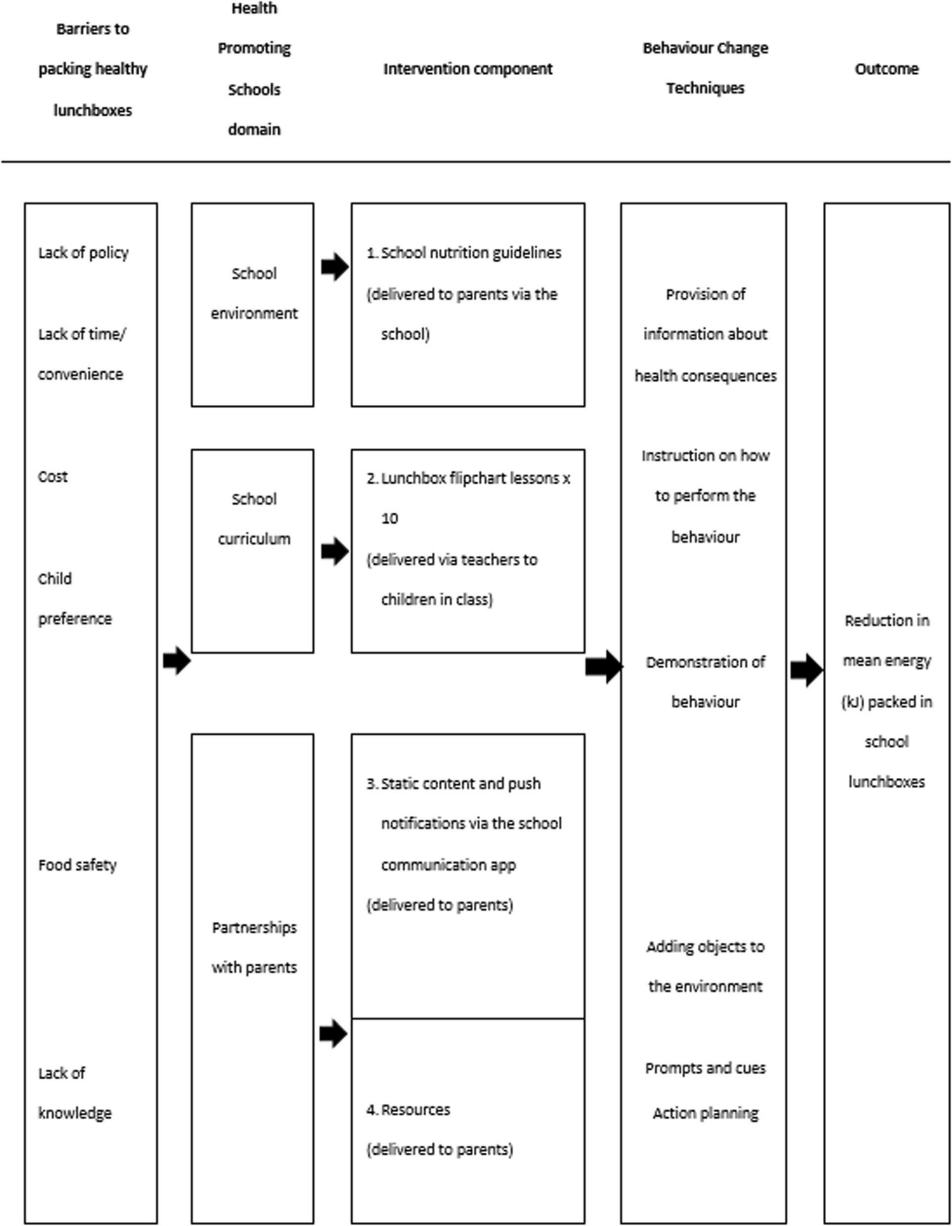
SWAP IT intervention logic

The BCW was used to select behavioural change techniques (BCTs) that were recommended to overcome the identified barriers to packing healthy lunchboxes. The BCTs were embedded in intervention components that spanned the domains of the HPS framework. The resulting logic model is presented in Fig. 2, and a detailed description of each component of the intervention is reported in Table 1.

**Table 1:** SWAP IT intervention components

Intervention component	Description
1. School nutrition guidelines	Schools received support to develop a school nutrition guideline outlining preferred foods to be packed in lunchboxes and guidance on how to limit the packing of discretionary food items. The guideline encouraged packing ‘recommended’ foods in the lunchbox every day in place of discretionary foods. Recommended items refer to foods and drinks from the core food groups as determined by the Australian Dietary Guidelines and Australian Guide to Healthy Eating [6]. Schools were provided with a template to assist in the development of the guidelines and encouraged to invite parents to assist with decisions regarding the content. The guideline was communicated to parents via school newsletter, school website and/or pushed to parents via the school mobile communication app prior to the second term of intervention. The school nutrition guideline addressed the identified barrier of lack of policy.
2. Lunchbox flipchart lessons	Schools and teachers were provided with a ten page flipchart for each classroom at the launch of the intervention. The flipchart features a different lunchbox sample for each week of the intervention and provides ideas for teachers to facilitate discussion on healthy lunchboxes in the classroom. The use of lunchbox flipchart lessons were designed to address child preference as a barrier to packing ‘recommended’ foods.
3. Parent communication pushed via a school mobile communication app (‘m-health’ component)	The intervention utilised an existing school mobile communication app (Skoolbag) to communicate lunchbox messages to parents/carers which address the barriers to packing a healthy lunchbox. Only active users of the school communication app were able to view the pushed intervention material. Therefore, in an effort to increase reach, parents were given instructions on how to download the school communication app at the beginning of the intervention period. In the first week of the second term of the SWAP IT intervention, eight static lunchbox themed pages (static content) were uploaded to the school mobile communication app. Additionally, parents received a push notification via the school mobile communication app once per week for 10 weeks (ten pushed messages in total). The static content and push notifications encouraged simple lunchbox swaps from common ‘discretionary’ foods to ‘recommended’ foods consistent with the dietary guidelines. Each push notification addressed a known barrier to packing healthy lunchboxes and included a “hook” (a headline designed to attract attention), pictures of lunchbox and swap examples, a 50–70 word message, a link to a video (only in selected pushes), link to the health organisation website housing additional content and an email address to request further information. For example, a pushed message may read: ‘(*Hook*) Veg-tastic lunchboxes: (*Pushed message*) Vegetables are packed with nutrients to help kids learn and play. And whilst it can be difficult to get kids to eat them you can make easy swaps to encourage greater vegetable intake at school. Set a goal to pack an extra vegetable item in your child’s lunchbox next week. For example: Swap out chips and sweet biscuits for vegetable sticks with hommus or salsa! For more great ideas to increase your child’s vegetable intake visit SWAP IT website. Once a message had been pushed to parents via the school mobile communication app, it appeared as static content on the school mobile app for parents to refer to at later stages when convenient.
4. Resources	In the first week of the SWAP IT intervention, each student received an information package containing tools and resources, including a lunchbox ideas booklet which provided easy, seasonal and low cost lunchbox ideas, ice-brick and ‘water only’ drink bottle to address the identified barriers of food safety, lack of time/ convenience, lack of knowledge, child preference and cost.

### Control schools

Control schools received either a physical activity intervention or no intervention (waitlist control). The physical activity intervention sought to increase primary school teachers scheduling of physical activity across the school week with the aim of meeting the recommended 150 min of planned moderate with some vigorous physical activity. Planned physical activity included time spent in PE, sport and other structured activities that is inclusive of all children and part of their regular programming and planning. Schools in the physical activity only and waitlist comparison schools were not offered the lunchbox intervention nor any other nutrition related support from the research team.

### Data collection procedures and measures

Baseline data was collected from principals, students and parents between February–March 2017, with the intervention occurring in August–October 2017 and follow up data collection undertaken immediately following the 10 week intervention in October–November 2017. The focus of this paper is on the nutrition outcomes of the intervention. The physical activity outcomes of the trial will be reported elsewhere.

### School and student demographics

School principals were asked to report the number of families enrolled at the school and confirm their postcode in order to classify school by location (urban or rural) and socio-economic status (SES) (high or low). Via the student consent form, parents were asked to report on their child’s sex, age (in years), postcode and whether they had downloaded the school mobile communication app. Students that resided in post-codes ranked in the top 50% of state post-codes based on the 2016 Socio-Economic Indexes for Australia (SEIFA) were categorized as ‘higher socio-economic areas’, whereas those in the lower 50% were categorized as ‘lower socio-economic areas’. Student’s post-codes were also used to categorize their locality as either ‘rural’ (those schools in outer regional, remote and very remote areas) or ‘urban’ (those in regional or major cities) based upon the 2016 Accessibility/Remoteness Index of Australia [32].

### Effectiveness of SWAP IT

The primary trial outcome was the mean kJ content of foods and beverages packed in children’s lunchboxes. Secondary outcomes included the mean total energy (kJ) from ‘recommended foods’ and percentage energy from ‘recommended foods’ packed in school children’s lunchboxes. To assess primary and secondary trial outcomes the content of children’s lunchboxes were analysed via observation (photograph). On a randomly selected school day, prior to recess, lunch and in-class vegetable and fruit breaks [33, 34], students were asked to display their lunchbox on their desk and remove all lids from containers. Trained research assistants took a photograph of each students’ lunchbox. Any lunchbox containers that identified the child were covered for the photograph. Photographs were analysed by trained Dietitians, blinded to group allocation at both time points. Dietitians assessed the nutritional content of lunchboxes using an electronic version of the School Food Checklist (SFC) [25, 35], a previously validated tool shown to be accurate and reliable in measuring energy from food and beverages for the Australian context. The SFC [25, 35] enables assessment of the kJ content and serving size for each lunchbox item. The checklist includes 20 food and beverage categories including main food items such as bread, fast food and leftovers/mixed dishes and snack items such as noodles, packaged snacks, biscuits and crackers, chocolate and lollies, cheese, eggs, dried fruit and nuts, muesli and fruit bars, cakes and buns, muffins and scones, pastries, desserts, yoghurt, fruit, vegetables, milk, soft drink, water and fruit juice. Foods in each category were included based on the frequency of consumption at school for children aged 5 to 15 years, according to the National Nutrition Survey 1995 [25] and the average kJ per category identified. The SFC was used to identify the total lunchbox contents and food items in the lunchbox including the kJ content, number of ‘recommended’ or ‘discretionary’ lunchbox items and the mean cost of lunchbox items. Recommended items refer to food and drink items that are part of the core food groups as determined by the Australian Dietary Guidelines and Australian Guide to Healthy Eating, including a wide variety of fruit, vegetables, grain (cereal) foods, lean meats and meat alternatives and dairy and dairy alternatives [6]. Food items classified as discretionary choices are items considered to be energy dense with minimal nutritional value such as cakes, chocolate, lollies, crisps, muesli bars and fast food.

Minor modifications were needed to the SFC to separately categorise recommended and ‘discretionary’ food choices and updated to reflect the mean cost of lunchbox items at the time the study was conducted (2017). Categories that required adjustment included: biscuit and crackers, cakes and buns, muffins and scones, desserts and packaged snacks. All foods in these categories were individually divided and categorised as a recommended or discretionary food by consensus among dietitians. The serve size and kJ per serve information was obtained from FoodWorks Professional Edition V7 (version 7; Xyris Software, Highgate Hill, QLD, Australia), or if unavailable from FoodWorks, via a snack food database created for pre-packaged items. The snack food database was created by Dietitians based on a significant array of pre-packaged snacks available in Australian supermarkets and included detailed nutrition information for each food item.

Dietitians were trained to classify foods and drinks according to their SFC category and the serving size of each item by observing each school lunchbox photograph. All lunchbox photos were assessed by two dietitians who worked together to make a consensus decision on the analysis for every lunchbox. To further aid this process, decision rules were developed to ensure standardisation of assessments. Differences in opinion between dietitians were resolved following consultation with a third assessor.

### Feasibility of SWAP IT

#### Delivery of intervention components to schools

Feasibility of delivering the non-app intervention components to schools were assessed via key informant interviews with school principals. Specifically principals were asked ‘Has your school developed school nutrition guidelines and communicated this guideline to parents?’ (yes/no) and ‘Did teachers use the lunchbox flipcharts?’ (yes/no).

#### Delivery of intervention components to parents (app engagement)

Feasibility of the app-based intervention components in reaching and engaging parents was assessed via analytic data assessing the number of pushed messages opened by parents and assessed via parent completion of a computer assisted telephone interview (CATI). Specifically, parents were asked: ‘Have you downloaded your school communication app?’ (yes/no), ‘Have you heard of the SWAP IT program?’ (yes/no), ‘Have you received lunchbox messages via the school communication app?’ (% of app users), ‘Did you receive a SWAP IT lunchbox ideas booklet?’ (yes/no), drink bottle for water only? (yes/no) and ice brick to keep your child’s lunchbox cold?’ (yes/no)?.

#### Self-reported behaviour change

Parents self-reported their behaviour change via completion of a computer assisted telephone interview (CATI) asking ‘Have you changed what you pack in your child’s lunchbox?’ (yes/ no, as I already pack healthy foods/ no).

### Acceptability of SWAP IT

#### Acceptability of the intervention to schools

Was assessed at a school level via key informant interviews asking the principal ‘Would your school like to continue with the SWAP IT program?’ (yes/no) and ‘Would your school recommend this program to other schools?’ (yes/no).

#### Acceptability of the intervention to parents

Acceptability of the intervention to parents was assessed via the CATI asking parents ‘Were the frequency of the messages you received via the app acceptable?’ (too frequent/ just right/ not frequent enough), ‘Were the SWAP IT messages helpful?’ (yes/no), ‘Is the school app the best way to get information to you?’ (yes/ no) and ‘Would you like to continue to receive similar messages?’ (yes/no). Parents were also asked the acceptability of the physical intervention resources including ‘Was the water bottle/ parent booklet/icebrick useful?’ (yes/no) and finally, ‘Was the SWAP IT website useful?’ (yes/no).

### Adverse outcomes

As encouraging families to provide healthy foods has been hypothesised to increase family financial burden [36], the mean cost of lunchbox items pre and post intervention were assessed via the SFC and were compared between intervention and control groups to determine if the intervention has caused any adverse financial effects for families. Costing was determined using an average of prices from food within the category accessed from local retail audit of similar foods determined in October 2016.

### Sample size

The sample was powered based on changes in mean kJ content of lunchboxes between groups. Assuming that a standard lunchbox contains 3087 kJ (SD = 1066 kJ) [13] and based on an ICC of 0.02, the participation of 150 students per school (with 6 schools per arm) would enable detection of a 205 kJ difference between groups at follow-up with 80% power at the 0.05 significance level.

### Analysis

All statistical analyses were performed using SAS (version 9.3) statistical software. All statistical tests were two tailed with an alpha value of 0.05. Summary statistics were used to describe all variables of interest.

Lunchboxes observations were considered valid for analysis if the student reported the lunchbox represented all food to be consumed at school. The lunchbox observation was excluded from analysis if a student indicated they would be purchasing a meal or snack from the canteen. As this 2X2 factorial study was designed for efficiency and was not powered to detect an interaction between the two intervention strategies, a conservative approach to analysis was taken whereby the main effects of each intervention strategy were analysed separately. However, the interaction between the two intervention strategies was explored as a sensitivity analysis, which was found to be non-significant.

Generalised Linear Mixed Models (GLMM), were used to assess trial primary and secondary outcomes related to mean kilojoules packed in lunchbox, kilojoules from recommended foods and percentage of kilojoules from recommended foods. All analyses were conducted under an intention to treat framework to test a mean difference between groups following the intervention, while adjusting for baseline values of the outcome and the physical activity intervention by including these outcomes as fixed effects in all models. Due to an imbalance in student SEIFA and remoteness classification between intervention and control groups, these variables were also controlled for in all models by including as a fixed effect in all models. A random level intercept for school was included to adjust for the clustered design of the study. All analyses were conducted on children whose parents had downloaded the school communication app at baseline given exposure to the parent component of the intervention required app access. The primary analyses were performed using all available data (complete case analysis), with a sensitivity analysis then being conducted using multiple imputation procedures for missing data [37]. Descriptive analysis was conducted to analyse the feasibility and acceptability data.

## Results

### Sample

Of the 12 consenting schools, the six schools allocated to the intervention group represented 1119 families. Parental consent was provided for 2143 students, of which baseline data was collected for 1915 (89%). Of the 2143 consenting students, 1552 parents also consented to the parent CATI (72%). The number of parents who then participated in the CATI was 948 at baseline (61%) and 802 (52%) at follow up. The characteristics of participating schools, students and parents at baseline was consistent across groups.

Of the 1915 lunchboxes that were observed at baseline, 1769 (94%) were assessed as valid, with 146 lunchbox observations excluded due to student’s indicating they would be purchasing from the canteen. The 1915 students represented 89% of students with parental consent. At follow up, 1462 student’s lunchboxes were observed, representing 68% of those with parental consent. Demographic information was collected via returned consent forms and parent consent calls. There was no significant differences in age, sex or socio-economic status between those who consented and those that did not consent. Baseline characteristics of the 12 schools and 1769 students are shown in Table 2.

**Table 2:** Sample characteristics of schools and students at baseline

School characteristics		
	Intervention	Control
Number of schools	6	6
Location		
• Urban	4	5
• Rural	2	1
School SES		
• Most disadvantaged	4	4
• Least disadvantaged	2	2
Number of schools greater that 10% Aboriginal or Torres Strait Islander student enrolments	1	2
	Intervention n (%)	Control n (%)
Total students	778	991
Sex		
• Female	379 (49.03)	480 (48.93)
• Male	394 (50.97)	501 (51.07)
Sex missing = 15		
Mean age (years)	7.99	7.94
Socioeconomic status		
• Most disadvantaged	574 (73.78)	648 (65.39)
• Least disadvantaged	204 (26.22)	343 (34.61)

Socioeconomic status (SES) based on SEIFA Index of relative socio-economic disadvantage. Most disadvantaged = lowest quartiles of SEIFA; Least disadvantaged = highest quartiles of SEIFA; SD, standard deviation

### Effectiveness of SWAP IT

Amongst parents, who reported downloading the school communication app (*n* = 1026), a non-significant reduction favouring the intervention group in the mean total energy of foods packed within lunchboxes was observed between groups (− 118.39 kJ, CI = -307.08–70.30, *p* = 0.22). Mean total lunchbox energy from recommended foods significantly increased in the intervention group (79.21 kJ, CI = -1.99, 156.43, *p* = 0.04) and percentage of lunchbox energy from recommended foods also increased in intervention schools, however was not statistically significant (4.86%, CI = -0.22, 9.95, *p* = 0.06) (Table 3). Analysis of data with complete cases showed consistent trends.

**Table 3:** Effectiveness of SWAP IT: Between group differences in mean total lunchbox energy, energy from recommended foods and percentage energy from recommended foods

Outcome	Intervention		Control		Imputed relative difference between groups at follow up^a^ Mean (CI)	*P* value	Complete case relative difference between groups at follow up^a^ Mean (CI)	*P* value
	Baseline Mean (SD) (*n* = 443)	Follow-up Mean (SD) (*n* = 373	Baseline Mean (SD) (*n* = 583)	Follow-up Mean (SD) (*n* = 487)				
Mean total lunchbox energy (kJ)	2691.85 (863.78)	2727.94 (920.14)	2704.60 (959.03)	2812.71 (959.03)	−131.61 [− 317.26–54.05]	0.16	− 133.32 [− 345.31–78.68]	0.19
Mean total lunchbox energy from recommended foods (kJ)	1578.62 (622.36)	1624.73 (568.76)	1607.40 (603.92)	1569.93 (591.01)	83.13 [2.65–163.61]	0.04	84.55 [−2.48–171.58]	0.06
Percentage of lunchbox energy from recommended foods	62.14 (24.09)	63.73 (23.89)	62.42 (24.24)	60.00 (23.91)	4.86 [−0.22–9.95]	0.06	5.50 [−0.28–11.29]	0.06

^a^The figures presented have been adjusted for baseline results

### Feasibility of SWAP IT

#### Delivery of intervention components to schools

All principals at intervention schools reported establishing school nutrition guidelines using the template provided and five were able to provide evidence of communicating these nutrition guidelines with parents. All principals’ schools reported using the curriculum flipcharts.

#### Delivery of intervention components to parents (app engagement)

A large proportion of parents reported downloading the school communication app (89%), approximately half of parents (46%) reported they had heard of the SWAP IT program and 71% recalled receiving the lunchbox messages via the school mobile communication app. The majority of parents reported receiving the intervention resources including the water bottle (96%), ice brick (92%) and parent resource booklet (90%).

Over the 10 week intervention period, a total of 372 additional app downloads were achieved, with a mean increase of 74 additional downloads per school. Across 1119 families represented within the intervention schools, app analytics identified the total views of the pushed messages ranged from 387 total views to 1550 views per week over the 10 weeks of messaging (indicating the content may have been viewed more than once by some families), with an average viewing rate of 1025 views per week. The week 1 message was the most viewed message with the Week 10 message regarding lunchbox ideas, being the least viewed (Table 4).

**Table 4:** Total views of the pushed messages via the school communication app during trial

Intervention content of pushed messages	Total Views
Pushed messages	
Week 1: Introduction	1550
Week 2: Budget	1266
Week 3: Time	1193
Week 4: Everyday foods	1133
Week 5: Tooth Decay	1091
Week 6: Food safety	1072
Week 7: Vegetables	1011
Week 8: Snacks	870
Week 9: Dairy	682
Week 10: Lunchbox ideas	387

#### Self-reported behaviour change

More than half of the parents (55%) self-reported that the SWAP IT program disseminated primarily via the school communication app changed what they packed in the school lunchbox.

### Acceptability of SWAP IT

#### Acceptability of the intervention to schools

All principals at intervention schools reported that they would like to continue to implement the SWAP IT program and would recommend the program to other schools.

#### Acceptability of the intervention to parents

The majority of parents (95%) considered the frequency of SWAP IT messages via the schools communication app to be just right, 84% reported the content was helpful and 68% agreed that the school communication app was the best way to receive lunchbox messages. In relation to physical resources, the majority of parents believed resources were very useful, including the water bottle (55%), parent resource booklet (57%) and ice brick (71%). Most parents also reported the SWAP IT website was useful (63%).

### Adverse outcomes

The mean total cost of the lunchboxes at baseline was $AUS3.72 (±1.27) and $AUS3.79 (±1.43) at follow up. There was no statistically significant difference in the cost of the total lunchbox between intervention and control groups at baseline ($AUS3.73 vs $AUS3.72, *p* = 0.73) or follow up ($AUS3.79 vs $AUS3.79, *p* = 0.40).

## Discussion

The SWAP IT pilot RCT is the first lunchbox trial internationally to evaluate the impact of an intervention delivered primarily via an existing school mobile communication app to reduce the overall mean energy of foods packed in the lunchbox, by targeting the replacement of discretionary foods with recommended foods. Results indicate the intervention is highly feasible, acceptable to both schools and parents, can be delivered with a high degree of fidelity and is potentially effective in reducing overall energy of foods packed in lunchboxes. Collectively, the findings suggest that the intervention may have public health merit and are supportive of a large RCT to establish the efficacy of the program.

There are few previous interventions that have examined the impact of a lunchbox intervention on energy intake [38]. While non-significant, the changes in overall energy of foods packed (− 118.80 kJ) were encouraging and consistent with the effects of dietary interventions targeting the availability of foods in school canteens [39] and of interventions conducted in other child food settings [40, 41]. At a population level, reductions in daily energy intake of 420 kJ have been estimated to be sufficient to prevent excessive weight gain in children [42]. While analyses of actual intake are required to assess whether the effects on the energy of foods packed for children translate into that consumed by children, the findings suggest that the intervention may make a meaningful contribution to population level energy reduction among children. The non-significant increases in the percentage of energy from recommended foods packed in lunchboxes (+ 4.86%) is also encouraging and suggests that the nutritional quality of foods packed for children has likely improved as a result of the intervention, and warrants an evaluation within a larger trial. Nonetheless, further research is required to substantiate these hypotheses.

Despite their potential, a criticism of app-based interventions to improve health behaviours is their limited capacity to access and engage their intended end-users. Even if identified and downloaded by end-users, 75% of apps [43] are deleted within 90 days, and open rates of push notifications are typically less than 10% [44]. This study found that 71% of parents recalled receiving the lunchbox messages and that pushed messages were viewed by 387–1550 (mean of 1025 views per week) over the 10 week intervention. Such findings suggests that embedding interventions in an existing mobile communication app used by schools can overcome some of the reported engagement barriers limiting the impact of app based interventions. However, views of push notification content reduced over time, in particular in the final weeks of the intervention. Ensuring that content most pertinent to packing healthy lunchboxes are introduced in early weeks of the intervention may improve its potential impact.

The findings suggest that the intervention may be well received by the school community. Sixty percent of schools approached agreed to participate in the study within a 2 week period signalling that a large proportion of schools may be amenable to adopting the intervention if offered as part of a government funded public health initiative. The implementation support strategies utilised in this study also appeared to be effective in ensuring that the broader school components were implemented as intended. Five of the six schools could provide evidence that nutrition guidelines were communicated with parents and over 90% of parents reported receiving the intervention resources. Such strategies could be considered by policy makers and practitioners interested in broader dissemination of the initiative if future trials establish its efficacy.

Strengths of the study include its experimental design, use of direct observation and validated tools to assess lunchbox contents and consideration of factors related to intervention acceptability and feasibility considered important in assessment of its potential real world utility. Nonetheless, the study findings should be considered in the context of its limitations. As a pilot trial, the study was not powered to detect clinically meaningful effects of the intervention, although it provides a rich source of data to inform the design of a larger RCT to establish its effects. The pilot also only included one follow-up assessment which occurred immediately post-intervention. The sustainability of the reported effect sizes are therefore not known, suggesting further longer term follow-up is required to determine if engagement with resources remains once the active intervention phase is completed. Potentially, any improvements in packing healthy lunchboxes, and child intake at school could be displaced through changes in intake occurring outside of school hours. Comprehensive assessment of dietary intake of children across the entire day is also required to examine the impact of the intervention on overall energy intake and subsequent child weight is therefore required. However, assessing dietary intake of young children is a considerable challenge. Young children cannot reliably report intake, and parents may be unaware of foods consumed at school. Different measure of dietary assessment, such as repeated lunch-box assessments prior to and following periods at school when children are provided time to eat to assess intake at school, supplemented with comprehensive parent completed dietary assessment for eating occasions outside of school hours may be required to robustly assess the impact of the intervention on student intake. Finally, complete cases analysis may result in a significant loss of data and consequently power due the reduced sample [45].

## Conclusion

Notwithstanding the limitations of the study, the research provides important information for researchers, policy makers and practitioners interested in the prevention of child obesity through school-based interventions. In particular, policy makers and practitioners urgently require suitable interventions likely to impact on energy balance, that have the ability to be easily implemented at scale. Future testing of this promising intervention will determine if the SWAP IT program represents such an intervention.

## Data Availability

The datasets analysed during the current study are available from the corresponding author on reasonable request.

## Abbreviations

BCT: Behaviour change techniques
BCW: Behaviour change wheel
CATI: Computer assisted telephone interview
HPS: Health promoting schools
kJ: kilojoules
SEIFA: Socio-Economic Indexes for Australia
SES: Socioeconomic status
SFC: School food checklist

## References

[1] Development Initiatives. Global nutrition report 2017: nourishing the SDGs. Bristol; 2017.

[2] Ng M, Fleming T, Robinson M, Thomson B, Graetz N, Margono C (2014). Global, regional, and national prevalence of overweight and obesity in children and adults during 1980–2013: a systematic analysis for the Global Burden of Disease Study 2013. Lancet.

[3] Dobbs R, Sawers C, Thompson F, Mankiya J, Woetzel JR, Child P, et al. Overcoming Obesity: An Initial Economic Analysis. Jakarta; 2014.

[4] World Health Organisation. Global Strategy on Diet, Physical Activity and Health: Childhood overweight and obesity: World Health Organisation; 2016. Available from: http://www.who.int/dietphysicalactivity/childhood/en/

[5] Hardy L, Mihrshahi S, Drayton B, Bauman A (2016). NSW schools physical activity and nutrition survey (SPANS) 2015: full report.

[6] National Health and Medical Research Council. Australian Dietary Guidelines. Canberra; 2013. Contract No.: 1864965754

[7] Chung A, Peeters A, Gearon E, Backholer K. Contribution of discretionary food and drink consumption to socio-economic inequalities in children's weight: prospective study of Australian children. Int J Epidemiol. 2018. 10.1093/ije/dyy020 29514246

[8] World Health Organisation. Health Promoting Schools: An effective approach to early action on noncommunicable disease risk factors. 2017 Available from: http://apps.who.int/iris/bitstream/handle/10665/255625/WHO-NMH-PND-17.3-eng.pdf?sequence=1. Accessed 17 Nov 2017.

[9] Evans CE, Greenwood DC, Thomas JD, Cade JE (2010). A cross-sectional survey of children’s packed lunches in the UK: food- and nutrient-based results. J Epidemiol Community Health.

[10] Hubbard KL, Must A, Eliasziw M, Folta SC, Goldberg J (2014). What’s in Children’s backpacks: foods brought from home. J Acad Nutr Diet.

[11] Bell AC, Swinburn BA (2004). What are the key food groups to target for preventing obesity and improving nutrition in schools?. Eur J Clin Nutr.

[12] National Health and Medical Research Council. Nutrient Reference Values for Australia and New Zealand including Recommended Dietary Intakes: dietary energy Australia: Commonwealth of Australia; 2006. Available from: https://www.nrv.gov.au/dietary-energy. Accessed 17 Nov 2017.

[13] Sanigorski AM, Bell AC, Kremer PJ, Swinburn BA (2005). Lunchbox contents of Australian school children: room for improvement. Eur J Clin Nutr.

[14] Horne PJ, Hardman CA, Lowe CF, Tapper K, Le Noury J, Madden P (2009). Increasing parental provision and children's consumption of lunchbox fruit and vegetables in Ireland: the food dudes intervention. Eur J Clin Nutr.

[15] Laurence S, Peterken R, Burns C (2007). Fresh kids: the efficacy of a health promoting schools approach to increasing consumption of fruit and water in Australia. Health Promot Int.

[16] Sweitzer SJ, Briley ME, Roberts-Gray C, Hoelscher DM, Harrist RB, Staskel DM (2010). Lunch is in the bag: increasing fruits, vegetables, and whole grains in sack lunches of preschool-aged children. J Am Diet Assoc.

[17] Nathan N, Janssen L, Sutherland R, Hodder RK, Evans CEL, Booth D, et al. The effectiveness of lunchbox interventions on improving the foods and beverages packed and consumed by children at centre-based care or school: a systematic review and meta-analysis. Int J Behav Nutr Phys Act. 2019;16(1):38. 10.1186/s12966-019-0798-1 PMC6489330 31036038

[18] Kipping RR, Jago R, Lawlor DA (2012). Developing parent involvement in a school-based child obesity prevention intervention: a qualitative study and process evaluation. J Public Health (Oxf).

[19] Payne HE, Lister C (2015). Behavioral functionality of mobile apps in health interventions: a systematic review of the literature. JMIR Mhealth Uhealth.

[20] Schoeppe S, Alley S, Van Lippevelde W, Bray NA, Williams SL, Duncan MJ (2016). Efficacy of interventions that use apps to improve diet, physical activity and sedentary behaviour: a systematic review. Int J Behav Nutr Phys Act.

[21] Grady A, Yoong S, Sutherland R, Lee H, Nathan N, Wolfenden L (2018). Improving the public health impact of eHealth and mHealth interventions. Aust N Z J Public Health.

[22] Reynolds R, Sutherland R, Nathan N, Janssen L, Lecathelinais C, Reilly K (2018). Feasibility and principal acceptability of school-based mobile communication applications to disseminate healthy lunchbox messages to parents. Health Promot J Austr.

[23] Janssen L, Sutherland R, Nathan N, Wyse R, Lecathelinais C, Finch M, et al. Parent acceptability of using a mobile phone application to promote healthy lunchboxes for childcare- and school-aged children. Currently under editorial review. 2018.

[24] Eldridge SM, Chan CL, Campbell MJ, Bond CM, Hopewell S, Thabane L (2016). CONSORT 2010 statement: extension to randomised pilot and feasibility trials. BMJ.

[25] Kremer PJ, Bell AC, Swinburn BA (2006). Calibration and reliability of a school food checklist: a new tool for assessing school food and beverage consumption. Asia Pac J Clin Nutr.

[26] Bandura A (2004). Health promotion by social cognitive means. Health Educ Behav.

[27] van Sluijs EM, McMinn AM, Griffin SJ (2007). Effectiveness of interventions to promote physical activity in children and adolescents: systematic review of controlled trials. BMJ.

[28] Lubans D, Morgan P (2008). Evaluation of an extra-curricular school sport programme promoting lifestyle and lifetime activity for adolescents. J Sports Sci.

[29] Baranowski T, Anderson C, Carmack C (1998). Mediating variable framework in physical activity interventions. How are we doing? How might we do better?. Am J Prev Med.

[30] World Health Organisation. Planning Meeting in Health Promoting Schools Project: Background, development and strategy outline of the Health Promoting Schools Project. Copenhagen; 1991.

[31] Michie S, van Stralen MM, West R (2011). The behaviour change wheel: A new method for characterising and designing behaviour change interventions. Implement Sci.

[32] Australian Bureau of Statistics (ABS) (2001). Technical Paper: Census of Population and Housing: Socio-Economic Indexes for Australia (SEIFA).

[33] Brennan L, Miles CL, Mitchell S, Matthews J (2010). Changes in the content of children’s school lunches across the school week. Health Promot J Austr.

[34] Nathan N, Wolfenden L, Butler M, Bell AC, Wyse R, Campbell E (2011). Vegetable and fruit breaks in Australian primary schools: prevalence, attitudes, barriers and implementation strategies. Health Educ Res.

[35] Mitchell SA, Miles CL, Brennan L, Matthews J (2010). Reliability of the school food checklist for in-school audits and photograph analysis of children's packed lunches. J Hum Nutr Diet.

[36] Rao M, Afshin A, Singh G, Mozaffarian D (2013). Do healthier foods and diet patterns cost more than less healthy options? A systematic review and meta-analysis. BMJ Open.

[37] White IR, Horton NJ, Carpenter J, statistics rim, social, Pocock SJ (2011). Strategy for intention to treat analysis in randomised trials with missing outcome data. BMJ.

[38] Evans CEL, Greenwood DC, Thomas JD, Cleghorn CL, Kitchen MS, Cade JE (2010). SMART lunch box intervention to improve the food and nutrient content of children's packed lunches: UK wide cluster randomised controlled trial. J Epidemiol Community Health.

[39] Wolfenden L, Nathan N, Janssen LM, Wiggers J, Reilly K, Delaney T (2017). Multi-strategic intervention to enhance implementation of healthy canteen policy: a randomised controlled trial. Implement Sci.

[40] Duncanson K, Burrows T, Collins C (2013). Effect of a low-intensity parent-focused nutrition intervention on dietary intake of 2- to 5-year olds. J Pediatr Gastroenterol Nutr.

[41] Hardy LL, King L, Kelly B, Farrell L, Howlett S (2010). Munch and move: evaluation of a preschool healthy eating and movement skill program. Int J Behav Nutr Phys Act.

[42] Cochrane T, Davey R, de Castella FR (2016). Estimates of the energy deficit required to reverse the trend in childhood obesity in Australian schoolchildren. Aust N Z J Public Health.

[43] Baidya A. Mobile app retention challenge: 75% users uninstall an app within 90 days (Report). 2016. Available from: https://dazeinfo.com/2016/05/19/mobile-app-retention-churn-rate-smartphone-users/. Accessed 23 Jan 2018.

[44] Accengage. 2017 Edition of the annual Push Notification Benchmark Accengage; 2017 Available from: https://www.accengage.com/press-release-accengage-releases-the-push-notification-benchmark-2017-including-for-the-first-time-web-push-facebook-messenger-metrics-in-addition-to-stats-for-mobile-apps/. Accessed 23 Jan 2018.

[45] Takkenberg JJM, Mokhles MM, Papageorgiou G, Grant SW (2018). Statistical primer: how to deal with missing data in scientific research?†. Interact Cardiovasc Thorac Surg.

